# A randomised trial of point-of-care tests for chlamydia and gonorrhoea infections in remote Aboriginal communities: Test, Treat ANd GO- the “TTANGO” trial protocol

**DOI:** 10.1186/1471-2334-13-485

**Published:** 2013-10-18

**Authors:** Rebecca J Guy, Lisa Natoli, James Ward, Louise Causer, Belinda Hengel, David Whiley, Sepehr N Tabrizi, Basil Donovan, Christopher K Fairley, Steven B Badman, Annie Tangey, Handan Wand, Mark Shephard, David G Regan, David Wilson, David Anderson, John M Kaldor

**Affiliations:** 1 The Kirby Institute, University of New South Wales, Sydney, Australia; 2 The Burnet Institute, Melbourne, Australia; 3 Baker IDI, Alice Springs, Northern Territory, Alice Springs, Northern Territory, Australia; 4 Apunipima Cape York Health Council, Cairns, Queensland, Australia; 5 Queensland Paediatric Infectious Diseases (QPID) Laboratory, Queensland Children’s Medical Research Institute, The University of Queensland, Queensland, Australia; 6 Department of Microbiology and Infectious Diseases, The Royal Women’s Hospital, Parkville, Victoria, Australia; 7 Department of Obstetrics and Gynaecology, The Royal Women’s Hospital and Murdoch Childrens Research Institute, University of Melbourne, Parkville, Victoria, Australia; 8 Melbourne School of Population and Global Health, University of Melbourne, Melbourne, Victoria, Australia; 9 Melbourne Sexual Health Centre, Melbourne, Victoria, Australia; 10 Ngaanyatjarra Health Service, Alice Springs, Northern Territory, Australia; 11 Flinders University International Centre for Point of-Care Testing, Flinders University, Adelaide, South Australia, Australia

**Keywords:** Point-of-care testing, Sexually transmitted infections, Randomized controlled trial

## Abstract

**Background:**

High prevalence rates of *Chlamydia trachomatis* (CT) and *Neisseria gonorrhoeae* (NG) have been reported in Aboriginal people in remote and regional areas of Australia for well over two decades, and repeat positivity rates are high. To interrupt disease transmission and reduce the risk of complications, early diagnosis and treatment is important. However in many remote and regional areas there are long delays between testing for these curable sexually transmissible infections and providing treatment, due to both physical distance from laboratories and difficulties when recalling patients for subsequent management once results are available. Point-of-care (POC) tests have the potential to provide more timely diagnosis, to increase treatment and contact tracing, and in turn reduce CT and NG infection rates.

**Methods/design:**

TTANGO (Test, Treat, ANd GO) is a cross-over cluster randomised controlled trial in 12 regional or remote Australian health services, which predominantly provide clinical services to Aboriginal people. The overall aim of TTANGO is to measure the clinical effectiveness, cost-effectiveness and cultural and operational acceptability of molecular POC testing for CT and NG infection. The primary outcome is repeat positivity at three months after treatment of an initial CT or NG infection.

Participating health services will undertake the clinical management of CT and NG under two different modalities for one year each. In the first year, six health services will be randomly assigned to manage these infections under current diagnostic guidelines. The other six will supplement current diagnostic guidelines with POC testing, whereby diagnosis is made and subsequent treatment for those with positive POC tests is offered at the initial consultation. In the second year, the health services will cross over to the opposite management modality.

TTANGO will be conducted over four years; 1.5 years of trial initiation and community consultation, 2 years of trial conditions and evaluation, and 6 months of data analysis and feedback.

**Discussion:**

TTANGO is the first cluster randomised trial of POC testing for CT and NG internationally. The results of this trial will provide crucial information to guide sexual health clinical practice in remote Aboriginal communities and other high prevalence settings.

**Trial registration:**

Australian and New Zealand Clinical Trials Registry ACTRN12613000808741

## Background

*Chlamydia trachomatis* (CT) and *Neisseria gonorrhoea* (NG) are sexually transmissible infections (STIs) that are fully curable with single dose treatment but are often asymptomatic for long periods of time [1]. Both infections can lead to serious complications [1] including pelvic inflammatory disease (PID) [2], ectopic pregnancy and tubal factor infertility [3,4], and a range of adverse pregnancy and neonatal outcomes [5,6]. The risk of PID increases by 4–6 fold in women with repeated CT infections [7].

Along with several other STIs, CT and NG have been reported at highly elevated rates in Aboriginal and Torres Strait Islander peoples (hereafter referred to as 'Aboriginal’) in Australia. According to the most recent national data, CT and NG were 3.6 and 30.6 times [8] more likely to be diagnosed in Aboriginal people, respectively, compared to the non-Indigenous population. As in many areas of health, the disadvantage experienced by Aboriginal people is amplified in regional and remote communities, where more Aboriginal Australian’s reside. Aboriginal people comprise approximately 2.5% of the total Australian population, yet 25% live in remote or very remote areas compared with < 2% of non-Indigenous people [9]. In 2011, CT and NG were diagnosed 8.3 and 61 times more often among Aboriginal people in remote areas, respectively, compared to non-Indigenous people in remote areas, and diagnoses rates were highest in young people aged 16–19 years [8]. Marked differences in rates of diagnosis among Aboriginal people are also evident according to area of residence; in 2011 CT and NG were diagnoses were 2.5 and 11 times higher in remote areas, respectively, compared to Aboriginal people in major cities [8]. Although the complications of CT and NG are not systematically monitored in Australia, clinical reports from remote Aboriginal communities in Central and Northern Australia indicate that PID and infertility occur at rates far higher than in other parts of Australia [10-12].

In regional and remote areas, primary health services play an important role in the prevention, testing, treatment and management of CT and NG. These health services are either State or Territory government funded or Federal government funded Aboriginal Community Controlled Health Services (ACCHS) and are the primary health care provider for Aboriginal people. There are an estimated 150 ACCHS located across Australia. Current clinical practice in most remote Aboriginal communities [13-15] follows guidelines which provide for immediate treatment if NG or CT are suspected on the basis of specified symptoms or risk [16]. Although symptom-driven diagnosis has the advantage of providing a rapid pathway to treatment, it has poor sensitivity and specificity for detecting infection, particularly in women [17,18]. Used as a diagnostic strategy on its own it therefore leaves many infections untreated, and results in unnecessary treatment in others.

As CT infections are mainly asymptomatic, and NG infections are mainly asymptomatic in women and in about 50% of men [19-21], treatment and contact tracing is usually initiated once a specimen has been tested and found positive by the laboratory. Treatment for these infections is almost inevitably delayed due to a range of factors including the time taken to transport the specimen from remote health services to laboratories [22], and delays in recalling patients when a positive diagnosis occurs. Some health services are more than 1000 kilometers away from the laboratory and pathology is sent on a weekly basis by small plane. A recent review of STI programs in remote Aboriginal communities showed that in a 12 month period only 75-89% of people diagnosed with an STI were treated, and the average time from diagnosis to treatment in asymptomatic people was 21 days [23] , far longer than the 2–3 day interval experienced in most urban settings [24].

In order to interrupt disease transmission, it is essential to diagnose and provide treatment as early as possible to reduce the average duration of infection among those with CT and NG [25]. Without treatment, infection with CT and NG can persist for on average 12 months and 6 months, respectively [26-28]. Timely diagnosis also reduces the risk of sequelae. A number of studies have found that within just a few weeks between testing and treatment, 2-3% of patients with CT infection have already developed PID [29,30]. Timely diagnosis also enables potentially infected sex partners to be contacted and treated (contact tracing), and in turn reducing the risk of re-infection in the index case which often occurs due to subsequent sex with an untreated existing partner [31]. Repeat chlamydial infections following treatment are also common. In a prospective cohort of young women in urban Australia, the repeat CT positivity rate was found to be 22.3% by 12 months [32]. Data based on routine re-testing remote Australian Aboriginal communities found the CT and NG repeat positivity rates was 50% within 12 months [33].

The use of point-of-care (POC) testing in the Australian Indigenous health sector is well established for chronic diseases (diabetes and renal diseases) through the national 'QAAMS’ (Quality Assurance for Aboriginal and Torres Strait Islander Medical Services) Program, which currently operates in over 170 Aboriginal medical services nationally [34,35]. POC tests for CT and NG would be the first use of infectious disease POC tests in this sector and could enable clinical services to offer treatment and begin the process of contact tracing at the initial consultation. However to date, use of CT and NG POC tests has been limited as only lateral flow devices have been commercially available with poor sensitivity and specificity [36,37]. Recently, molecular CT and NG POC tests have been developed, which have very high sensitivity and specificity of >95%, providing an opportunity to utilize these tests to control infections rates in high prevalence settings [38]. Mathematical modeling by Hui *et al*. [39] has shown that CT/NG POC tests with 95% sensitivity could reduce the prevalence of CT in remote Aboriginal communities from 11.9% to 8.9% and NG from 7.1% to 5.7% and if the annual STI screening coverage is increased from 44% (estimated current rates) to 60% the prevalence of the infections could drop further to 3.1% and 1.8% for CT and NG, respectively.

The overall aim of the TTANGO trial is to measure the clinical effectiveness, cost-effectiveness and cultural and operational acceptability of POC testing for CT and NG infections in remote Aboriginal communities. The primary objective is:

1. To determine whether the addition of POC testing to standard diagnostic procedures significantly decreases rates of repeat positive infections at three months among people with CT or NG infection in remote communities.

The secondary objectives are to determine:

1. Whether the addition of POC testing to standard diagnostic procedures significantly increases the proportion of CT and NG infections treated within seven days of specimen collection;

2. The cultural acceptability of POC tests to patients.

3. The operational acceptability to health service staff in remote community settings.

4. The cost effectiveness of the addition of POC testing to standard diagnostic procedures in remote primary health care services.

5. The diagnostic performance characteristics (sensitivity, specificity and positive and negative predictive value) of the POC test in a field setting compared to conventional laboratory testing.

6. A best practice model for quality management of STI/POC testing in remote health services.

## Methods

### Study design

**Figure 1:**
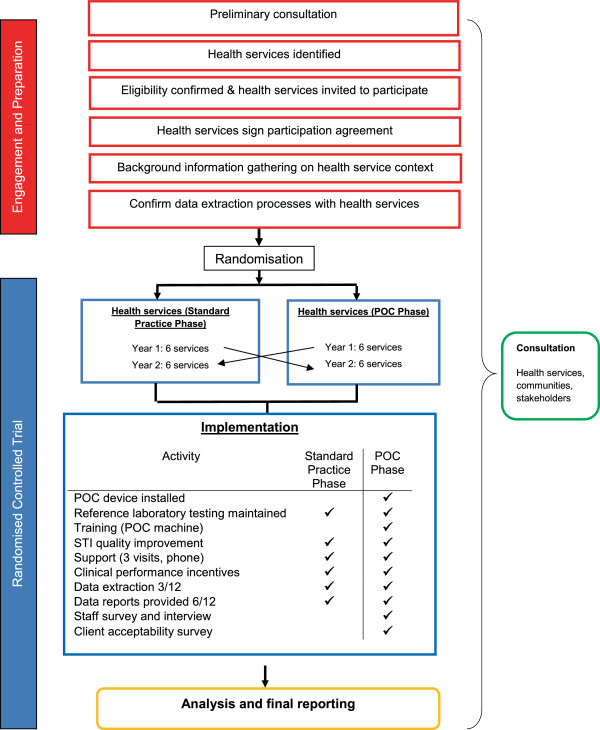
Trial overview.

TTANGO is a crossover cluster randomised controlled trial set in 12 health services in regional or remote areas of Queensland, West Australia and South Australia (Figure 1: Study overview). Participating health services will undertake the clinical management of CT and NG under two different parallel modalities for one year each, in a randomly assigned order. In the first year, six of the health services will be randomly assigned to manage these infections under current diagnostic guidelines (“standard practice” phase or “control” phase). The other six will supplement current diagnostic guidelines with POC testing (“POC” phase), such that diagnosis is made and subsequent treatment for those with positive POC tests is offered at the time of initial consultation. In the second year, the health services will cross over to the opposite management modality. The study will require four years to complete, including 1.5 years preparation and community consultation, 2 years for the intervention and evaluation, and 6 months for analysis and reporting. The fundamental premise of the trial is that more timely treatment for STIs (both index case and partners), will result in a substantial reduction in the percentage of people with repeat positive CT and NG infections.

A randomised trial is required because it will provide the most compelling evidence to guide future use of a novel technology which is currently not routinely used for STI management. The cross-over design allows each health service to act as its own control, thereby eliminating the effect of between-health service variation in the study endpoints. Furthermore, health services are more inclined to participate in a trial of an innovative approach to service delivery if they are guaranteed to receive the intervention during the course of the trial. Individual patient randomisation would not be feasible as the POC tests would then be used for some patients but not others within the same health service, potentially causing disruption and confusion for health service staff and patients.

CT and NG are the focus of the trial, because they occur at very high rates in remote Aboriginal communities and are easily detected and curable with single dose treatment, making them good candidates for strategies which aim to improve detection and treatment of infections with antibiotics. Although syphilis is also curable and has been endemic in some remote communities, rates have decreased considerably over the past decade and is now much rarer than the other STIs [8]; also at the time of the trial design syphilis point-of-care tests were unable to differentiate recent from past syphilis infection [40]. Despite its focus on CT and NG, TTANGO encourages best practice for the detection and treatment of all STIs.

### Ethics and informed consent

The TTANGO study protocol has been approved by five ethics committees from participating jurisdictions: the Western Australian Aboriginal Health Information Ethics Committee, the Western Australian Country Health Service Board Research Ethics Committee, the Cairns and Hinterland Health Service Human Research Ethics Committee, the Townsville Health Service District Human Research Ethics Committee and the Aboriginal Health Research Ethics Committee of South Australia.

Participating health services sign a Site Participation Agreement prior to commencement of their involvement in TTANGO. This agreement outlines trial details, responsibilities of the research team and participating services, principles of confidentiality and data ownership, and defines processes for reporting of findings and public release of study results.

### Community engagement and collaboration

Engagement with stakeholders including Government, state-based Aboriginal Community Controlled Health Organisations (which represent ACCHSs in each state), and laboratories will be a key principle of the TTANGO trial. Effective community engagement will ensure that an appropriate framework is developed to guide project implementation, and that transparency is maintained at all levels.

### Identification of communities and health services and eligibility criteria

In the first instance, potentially eligible health services will be identified in consultation with Government and Aboriginal Community Controlled Health Organisations. Identified health services will then be approached and invited to participate in TTANGO if they meet the following inclusion criteria: located in communities classified as regional, remote or very remote by the Australian Bureau of Statistics [41]; annually offer CT and NG testing to a minimum of 150 people aged 16–29 years (see sample size section), with a CT and NG positivity of at least 10% in 16–29 year olds; agree to be randomly assigned in one year out of two to the POC phase; have an electronic patient management system; and have the staff and resource capacity to fulfil protocol requirements.

### Randomisation

Health services within the four geographic regions will be randomised to intervention (“POC” phase) or control arm (“standard practice” phase) strategies for year one in a 1:1 ratio, and will cross over to the opposite arm in year two. Randomisation within geographic regions is mainly for pragmatic reasons so that the TTANGO coordinators are able to manage workload appropriately, and avoid the possibility of all services in one region being randomised to the same study arm. The randomisation scheme will be developed by the TTANGO statistician using SAS statistical software (Version 9.3) and will be communicated to participating health services once participation agreements have been signed.

During the ***POC Phase*** health services will continue routine STI testing and management practices (syndromic management, and sending off specimens to laboratories for routine NAAT tests) but will supplement their activity with CT and NG POC testing, so treatment and management of positive CT and NG POC tests occurs at the time of initial consultation. The POC test selected for the trial is the GeneXpert CT/NG® (Cepheid) which is a new test based on nucleic acid amplification technology, with dual detection of CT and NG in under 90 minutes. The GeneXpert CT/NG POC test had been demonstrated in laboratory and field evaluations in Australia [42,43] and the US [44] to have similar sensitivity and specificity to routine laboratory based NAAT tests, and was easy to use. The test has 5 targets, including a specimen processing control, specimen adequacy control, one CT target and two targets for NG. Two separate targets for NG allows for more specific detection and confirmation of NG [45]. The GeneXpert CT/NG test was approved by the Therapeutic Goods Administration for diagnostic purposes in Australia in March 2013, and obtained Conformité Européenne (CE) marking and US Food and Drugs Administration (FDA) clearance in 2012.

During the ***Standard Practice phase*** health services will maintain routine STI testing and management practices involving syndromic management, and sending off specimens to laboratories for routine NAAT tests, with treatment and management of asymptomatic infections based on the results that are received from the laboratory. The NAAT tests commonly used at the laboratories are the Roche Cobas and the Gen Probe Aptima.

Health services in both trial phases will receive *clinical performance incentives* and will participate in *STI quality improvement* (see further details below). Health services operating in the POC Phase will receive *POC training and ongoing support* and will participate in *laboratory quality management procedures* (see below).

#### STI quality improvement

In both trial phases, de-identified clinical and laboratory data will be used to generate regular site specific reports on STI screening and management. Reports will show the percentage of health centre attendees patients tested for STIs, the percentage testing positive, time to treatment, the percentage re-tested 3 months after treatment, and the percentage with a repeat positive result at re-test. The reports will be discussed at initial and subsequent visits, and are intended to assist services to reflect on current practice and develop strategies to achieve best practice in STI service delivery [16,46,47]. Accordingly, health services will be supported to increase and appropriately target opportunistic STI testing, provide treatment promptly where indicated, and follow recommended guidelines for re-testing and contact tracing. Where necessary, TTANGO coordinators will respond to identified training needs to strengthen sexual health service delivery.

#### Clinical performance incentives

In both trial phases, health services will receive incentive payments at six monthly intervals. Payments will be slightly higher in the POC phase, in recognition of the additional time spent fulfilling trial data collection requirements. Over the course of the trial (POC phase and Standard Practice phase), health services will be eligible to receive a maximum of $5000. In the POC phase, payments will be on the basis of $20 per POC test, up to a maximum of 150 tests, and $30 per re-test in clients who test positive. In the standard practice phase, payments will be on the basis of $10 per test, up to a maximum of 150 tests, and $30 per re-test in clients who test positive. Trial coordinators will provide support in both phases of the trial to strengthen testing and re-testing practices.

#### POC training and support

In the POC phase, health service personnel will be trained onsite in use of the GeneXpert machine, complete a competency assessment, and will be provided with all materials necessary to perform POC testing throughout the POC phase. 'Real-time’ monitoring and support will be provided remotely by TTANGO coordinators, through the use of 'LogMeIn’ software. Trial coordinators will also provide support at their 6-monthtly visits and by phone/email.

#### Quality management for POC processes

The trial will incorporate Quality Management procedures to ensure that QM is conducted and that data are generated, documented and reported in compliance with good clinical laboratory practice. Standard operating procedures (SOPs) for POCT have been developed and are reinforced through POCT training and competency assessment processes, and compliance with SOPs is monitored during site visits. Any specimens generating discordant results (between laboratory reference test and the GeneXpert) will be stored at the local laboratory, and at the end of the trial transferred to a research laboratory for re-testing with the COBAS 4800 test. Whilst the GeneXpert machine has a range of sophisticated built-in quality test processes, health services will be required to test a known positive “in house” quality control sample (consisting of non-infectious DNA of both CT and NG and prepared to simulate an infection of average bacterial load) each month and four blinded external quality assurance samples twice during the trial. The quality assurance procedures were developed by the National Serology Reference Laboratory for this trial and involve sending out swabs with freeze-dried organism (CT, CT and NG, NG and negative) [48].

### Outcome measures

The primary outcome is repeat positivity at three months after treatment among people with CT or NG infection.

The secondary outcomes are:

1. Proportion of infections treated within seven days of specimen collection.

2. Cultural acceptability of POC tests to patients.

3. Operational acceptability of POC tests to health service staff in remote community settings.

4. The cost effectiveness of the addition of POC testing to standard diagnostic procedures.

5. The sensitivity, specificity, negative and positive predictive value of the POC test in a field setting compared to the reference laboratory test.

6. A best practice model for quality management of STI/POC testing in remote health services.

### Data collection methods and variables

The trial will utilise a number of methods to collect information to calculate the trial outcomes above and these are described in further details in Table 1. Confidentiality will be maintained at all levels of data collection, transfer and analysis by de-identifying participating health services, staff, patients and other key stakeholders.

**Table 1:** Tools, methods and description of information collected in the trial

**Tool**	**Method and frequency**	**Description of data collected**
Site assessment tool	• Completed by TTANGO Coordinators at baseline, and updated 6 monthly in all health services.	*Broad description of*:
		(i) Clinical services and staffing
		(ii) STI testing practices
		(iii) Patient information system
		(iv) Clinic setting as it relates to location of GeneXpert machine and consumables
Clinical data (quantitative)	• Collated from one or more of the following sources:	(i) Patient consultation data and associated demographics
	(i) Health service patient information management systems	(ii) STI testing (point of care and laboratory), retesting and treatment outcomes
	(ii) Laboratory data	
	• Extracted 3 monthly for all health services	
Quality management data	• Services record results of EQA and forward to coordinators,	• External quality assurance and internal quality control test results
	• twice during POC phase	• Temperature range of consumables during transport
	• Internal quality control testing, once per month	• Multiple choice quiz and observed practice against competency standards
	• Temperature monitoring of test consumables during transport	
	• Knowledge and competency assessment of staff following training in GeneXpert usage	
Surveys (quantitative)	• Staff acceptability survey at end of POC phase	• Confidence using the test; satisfaction with training; trust in the results; ease of use; experience of discordant results; and impact on health service operations.
	• Client acceptability survey, with attending clients in last month of POC phase	
		• Preference for testing modality (POC test vs conventional); convenience; satisfaction with test process and communication of results
In-depth interviews (qualitative)	• Stakeholders (national)	• Challenges and implications of introducing POC testing
	• Stakeholders (national and international)	• Quality management and training considerations
		• Acceptability
	• Health service staff	

### Sample size

Power calculations were performed using the methods of Julious et al. [49], for paired binary comparisons and Hayes et al. [50] for community randomised trials. We assumed that the prevalence of CT and/or NG will be 15% at recruitment to the trial in each year, that 30% of patients will have a positive re-test within three months (in the standard practice phase), and that at least 150 patients aged 16–29 years will be tested at each health service in both POC and standard practice years. Table 2 shows the total number of health services needed to detect a reduction in CT and NG repeat positivity rate from 30% in control health services to 15% in intervention health services with 80% and 90% power, differing community coefficients of variation (0.25, 0.30) and equal numbers of health services in each arm. At least ten services will provide data in both the intervention and control arms. This will ensure 88% power to detect a decrease in CT and NG repeat positivity rate from 30% to 15%, and 78% power to detect a reduction from 20% to 10%. Conservatively, 12 services will be included in the trial.

**Table 2:** Sample size estimates based on reduction in repeat positivity rate from 30% to 15%

**Coefficient of variation**	**Number of services**	
	**80% power**	**90% power**
25%	8	10
30%	10	14

### Statistical analysis

We will compare results between the two years (with and without POC testing) from within the 150 patients aged 16–29 tested each year in participating health services. Characteristics of participating health services will be summarized at baseline and across trial arms (standard practice or POC phase). The primary endpoint of the study will be the health service specific difference between the two years in repeat positivity rates at three months. The secondary endpoint will be the health service specific difference between the two years in the proportion of true CT and NG infections that were treated within seven days of presentation. For both the primary and secondary endpoints, the average difference between the two years (with and without POC tests) will be calculated across participating health services. Other secondary outcomes will be measured as follows:

*Cultural acceptability of POC tests to patients*: will be assessed by a descriptive analysis of questionnaire feedback received from patients.

*Operational acceptability of POC tests to health service staff in remote community settings*: will be assessed by calculating the proportion of staff who indicated that they agreed or strongly agreed on the Likert scale with the acceptability questions in the staff questionnaire.

*The cost effectiveness of the addition of POC testing to standard diagnostic procedures*: The treatment interval, repeat positivity rate and contact tracing data collected in this trial will enable us to parameterise the models. Our analyses will apply similar techniques to those used by other studies in the context of POC tests [51,52], but explicitly represent regional and remote Australian Aboriginal community settings. Our approach will extend previous work by developing a more realistic model based on simulated sexual networks rather than population averages. The mathematical approach will use an individual-based model to enable us to accurately simulate and track repeat positivity rates, which is not possible with previous models. The epidemiological transmission models will project the estimated prevalence, incidence and repeat positivity rates expected from implementing POC tests in communities.

These model outputs will become inputs in a health economics analysis. The cost of using POC tests in regional and remote health services including the increased clinician and patient waiting time for POC tests and the patient costs incurred in a return visit to the health service for medication after a positive test result will be directly measured in the trial. These data will be used to estimate the additional cost of implementing POC tests in combination with NAAT testing, compared to standard diagnostic procedures. Costs related to research will be excluded.

The total costs associated with the widespread implementation of POC tests, including estimated logistical, clinical and administrative costs, will be compared with the long-term health and financial savings associated with their use (including the prevention of new infections, co-morbidities, and saving in reduced quality of life as well as costs of treatment saved due to decreased infections). As is standard with health economic analyses, discounting will be applied at levels of 0%, 3% and 5%. The health economic analyses will be carried out from the perspectives of the provider and the client. The integrated epidemiological and economic modelling will examine issues of allocation, efficiency, and cost-effectiveness.

*The sensitivity, specificity, negative and positive predictive value of the POC test in a field setting compared to the reference laboratory test*: POC test performance will be assessed by comparing the results of the POC tests performed during the POC phase with the reference test (NAAT).

## Discussion

Rates of CT and NG in remote Aboriginal communities in Australia are unacceptably high, and TTANGO will evaluate whether POC testing for these STIs can improve the timeliness of treatment and in turn reduce repeat infection rates. To our knowledge, TTANGO will be the first randomized trial of molecular POC tests for CT/NG infection internationally.

It is anticipated that one of the main challenges for TTANGO will be to sustain the involvement of health service staff amidst the numerous competing demands and health priorities, and also ensure POC operator competency is maintained in the context of high staff turnover. Regular communication and training with trial sites from a dedicated TTANGO coordinator should help to mitigate this risk. Another challenge will be if the services randomised to the POC arm in the first year prefer to keep the POC machines due to realized improvements in STI management. In the consultation phase we emphasized to health services the importance of the cross-over design to ensure long term policy change and funding. Finally, repeat positivity rates are calculated from those who re-tested and these need to be similar in both POC and non-POC arms to minimize biases – lower re-testing rates are often associated with higher repeat positivity due to re-testing in those with symptoms [53]. To mimimise any imbalance in re-testing rates, a quality improvement program and incentives associated with re-testing have been provided in both arms.

This research is significant as study outcomes will make an important contribution to the international understanding of STI control using POC tests. The research may also lead to significant decreases in STI prevalence and reinfection across multiple sites with long term benefit for the sexual health and wellbeing of young Aboriginal people. Finally, it has the potential to influence public health policy and result in [54] uptake of CT and NG POC tests into further remote clinical services in Australia, and other settings.

## Competing interests

No financial support was received by Cepheid. Cepheid has provided GeneXpert machines on loan for the duration of TTANGO.

## Authors’ contributions

RG and LN prepared the manuscript. All other authors reviewed the manuscript and contributed to the research protocol. All authors read and approved the final manuscript.

## Pre-publication history

The pre-publication history for this paper can be accessed here:

http://www.biomedcentral.com/1471-2334/13/485/prepub
